# Disseminated BCG: Complications of Intravesical Bladder Cancer Treatment

**DOI:** 10.1155/2014/362845

**Published:** 2014-06-12

**Authors:** Uyen To, Joyce Kim, David Chia

**Affiliations:** Yale School of Medicine, 333 Cedar Street, New Haven, CT 06510, USA

## Abstract

Intravesical bacillus Calmette-Guerin (BCG) has been established as an effective treatment of superficial bladder cancer (Parker and Kommu, 2013). However, major side effects, including pneumonitis, sepsis, and even death, may occur in <5% of patients (Gonzalez et al., 2003). Here we present a case of severe disseminated *Mycobacterium bovis* following intravesical BCG administration. Our patient is a 76-year-old gentleman with newly diagnosed superficial transitional cell carcinoma of the bladder who recently received his first intravesical BCG treatment. He initially presented with hemoptysis and was found to have extensive patchy infiltrates bilaterally. He was treated for pneumonia with antibiotics and then with steroids for hypersensitivity pneumonitis but continued to deteriorate. Due to the temporal proximity of his exposure to BCG, we administered treatment for presumed disseminated BCG infection with rifampin, isoniazid, and ethambutol. Within a 48-hour period, the patient improved dramatically. The reported cases of infection from intravesical BCG illustrate an insidious onset with initial symptoms of low-grade fevers and cystitis but may progress to pneumonitis. If the symptoms persist for more than 7 days or if there is clinical deterioration, RIPE therapy (with rifampin, isoniazid, pyridoxine, and ethambutol) and a fluoroquinolone should be administered for a 6–9-month course along with steroids for 4–6 weeks (Naudžiunas et al., 2012).

## 1. Introduction

Intravesical bacillus Calmette-Guerin (BCG) has been established as an effective alternative to standard chemotherapeutic regimens for the treatment of superficial bladder cancer [1]. BCG is a live-attenuated strain of* Mycobacterium bovis*, which induces a cell-mediated immune response, triggering an attack against malignant cells [2]. This approach is effective, with 60–70% of patients achieving remission. However, approximately 30–80% of patients can experience recurrence within 5–10 years after treatment [3, 4]. Overall, the treatment is well tolerated. When side effects do occur, they are generally mild, including fever, nausea, hematuria, and dysuria. However, in <5% of patients, intravesical BCG administration can result in severe complications, including pneumonitis, hepatitis, sepsis, and even death [3]. Here we present a case of severe disseminated* Mycobacterium bovis *following intravesical BCG administration and discuss the clinical risk factors and relevant treatment options for this rare adverse event.

## 2. Case History

Our patient is a 76-year-old gentleman with a past history significant for remote lung cancer status after left posterior lower lobectomy, prostate cancer status after prostatectomy, atrial fibrillation on anticoagulation and tachy-brady syndrome status after pacemaker placement, stroke with residual weakness, and newly diagnosed superficial transitional cell carcinoma of the bladder who recently received his first intravesical BCG treatment. He initially presented to our hospital with a complaint of hemoptysis. The patient reported that over the last two days he developed a productive cough with yellow sputum. On the day of admission, he noticed streaks of blood within the mucus itself, prompting his presentation to the emergency department.

On review of systems, he denied any fevers, chills, weight loss, night sweats, substernal chest pain, shortness of breath, nausea, vomiting, dysuria, hematuria, recent urinary tract infection, urinary catheterization, hematochezia, melena, recent travel, or sick contacts. His medications were notable for warfarin and amiodarone for atrial fibrillation. His social history was notable for a 30-pack-year smoking history and exposure to asbestos during his previous employment as a construction worker.

In the emergency department, his initial vital signs included a temperature of 97.8, heart rate of 77, blood pressure of 145/69, and oxygen saturation of 92% on 5 liters of oxygen via nasal cannula. On physical exam, he was a well-appearing man in no acute distress. He had no lymphadenopathy. His heart exam was only notable for an irregularly irregular rhythm. His lung exam was significant for right lower lung rales, but no wheezing or rhonchi. His abdomen was benign, skin was absent of any lesions, and extremities were notable for clubbing. Initial laboratory testing was unremarkable with a normal complete blood count (WBC 8.3) and normal comprehensive metabolic panel. A chest X-ray showed extensive infiltrates throughout both lungs. Given his hemoptysis and hypoxia, a CT scan of the chest was obtained to evaluate pulmonary embolism, which revealed extensive patchy infiltrates throughout the right lung and left lower lung base, without evidence of pulmonary embolism. Blood, urine, and sputum cultures were obtained and he was empirically treated with vancomycin and ampicillin/sulbactam.

Within 48 hours of hospitalization, his hypoxia progressed to the point of requiring 100% oxygen via nonrebreather. He subsequently completed a seven-day course of ceftriaxone and doxycycline for presumed community acquired pneumonia with no improvement of his symptoms. Given his clinical deterioration, despite appropriate antibiotic treatment, his warfarin was held due to concern for possible diffuse alveolar hemorrhage, amiodarone was discontinued for concern of amiodarone induced pulmonary toxicity, and high dose IV steroids were initiated for possible hypersensitivity pneumonitis. Additional fungal and AFB cultures were also obtained. All cultures returned negative, except for sputum and urine AFB cultures. These were positive for* Mycobacterium avium intracellulare*, which were determined by the infectious disease service to be contaminants.

He remained oxygen dependent on a nonrebreather despite high dose steroids. Given no perceived benefit of this treatment, the steroids were gradually tapered off, without any worsening of his clinical condition. In order to ascertain a definitive diagnosis and to evaluate the possibility of disseminated BCG infection, a video-assisted thoracoscopic lung biopsy was performed. Pathologic exam revealed chronic lung fibrosis with evidence of ossification. These findings did not explain his acute hypoxic respiratory failure. Due to the temporal proximity of his exposure to BCG and failure of all other therapies, we initiated empiric treatment for disseminated BCG with rifampin, isoniazid, and ethambutol. Within a 48-hour period, the patient's oxygenation improved dramatically. Subsequently, he was discharged home on 2 liters/min of oxygen to complete a 9-month course of treatment.

## 3. Discussion

Since the first study published by Morales et al., BCG intravesical installation has emerged as an effective treatment for nonmuscle invasive bladder carcinoma [3]. Though generally well tolerated, our case illustrates that devastating side effects may occur.

The pathophysiology of disseminated BCG in this setting is still unclear [2]. In an intact immune system, macrophages in the bladder take up the mycobacterial antigen and present it to T helper cells, which then triggers a cascade of interactions between MHC I, lymphocyte function antigen I, IL-1, IL-2, CD-28, and CD-80. This leads to a hypersensitivity reaction within the mucosal surface of the bladder that gives BCG its antitumor effect [2, 5]. Theoretically, those who lack cellular immunity cannot mount this response, thereby allowing* Mycobacterium bovis* to disseminate [5]. Alternatively in immunocompetent hosts, dissemination of the pathogen likely occurs secondary to trauma or inflammation permitting the organism to enter the bloodstream [5].

In order to prevent complications of intravesical BCG, patients who are pregnant or immunosuppressed should not receive the treatment [6]. Since BCG is a live-attenuated strain of* Mycobacterium bovis*, intravesical treatment and vaccinations should both be avoided in this population [2]. Of note, pediatric cases of disseminated infection have been reported after administration of the BCG vaccination in those with congenital immunodeficiencies [7].

In addition, patients with active systemic or urinary tract infections, gross hematuria, recent bladder biopsy, transurethral resection of prostate (TURP), or traumatic catheterization should have their instillation deferred until a safer time [6].

Infection from intravesical BCG generally follows an insidious course, with presenting symptoms occurring between one week and several months after exposure [6]. Initially, these symptoms may include low-grade fevers, chills, malaise, weight loss, arthralgias, nausea, vomiting, pneumonitis, hepatitis, and lower urinary tract symptoms. Later complications may include localized disease in the genitourinary tract (including testicular mass or prostatitis), osteomyelitis, mycotic aortic aneurysm, and cytopenias [8, 9]. After multiple installations of intravesicular BCG, granuloma formation may also occur [6]. Unfortunately, differentiating disseminated infection from hypersensitivity reactions can be difficult, given significant overlap in symptomology [5]. Rarely have fulminant cases occurring within 12 hours of instillation been reported. These are often associated with high fevers (>39°C) suggestive of a rapidly progressing infection and portend dismal outcomes with a 50% mortality [6].

A review of our patient's case revealed no specific risk factors that would predispose him to disseminated BCG, including immunosuppression, recent urinary tract infection, or hematuria. However, his presentation exemplified the difficulty of diagnosing disseminated BCG, including the insidious nature of the disease and the nonspecific symptoms of chills, malaise, and low-grade fevers. This presentation demonstrates the critical importance of maintaining a high level of suspicion for disseminated BCG, as early diagnosis and treatment are imperative for preventing morbidity and mortality.

In terms of management, patients undergoing BCG installation who develop low-grade fevers and symptoms of cystitis should be hospitalized and closely monitored [5]. If symptoms are mild, treatment with antibiotics to cover Gram-negative organisms can be administered while the patient is observed [2, 5]. If symptoms are more severe, empiric isoniazid may be initiated. If symptoms persist for more than 7 days, or if there is evidence of clinical deterioration, RIPE therapy (rifampin, isoniazid, pyridoxine, and ethambutol) and a fluoroquinolone should be administered for a 6–9-month course with steroids for 4–6 weeks in order to definitively treat disseminated BCG infection [5].

Currently, there is no reliable laboratory test available to confirm the diagnosis of disseminated BCG infection. Although the yield is low, blood and urine AFB cultures should be obtained [2]. Tissue biopsy and culture should be performed to evaluate noncaseating granuloma formation and presence of* Mycobacterium bovis*, respectively [2]. However, tissue cultures also have low yield, with prior studies demonstrating positive results in only 30% of cases [2]. As a result, a high clinical suspicion is critical in order to prevent delays in treatment initiation [6].
